# Plasma kallistatin levels in patients with severe community-acquired pneumonia

**DOI:** 10.1186/cc12507

**Published:** 2013-02-08

**Authors:** Wei-Chieh Lin, Shiou-Ling Lu, Chiou-Feng Lin, Chang-Wen Chen, Lee Chao, Julie Chao, Yee-Shin Lin

**Affiliations:** 1 Medical Intensive Care Unit, Department of Internal Medicine, National Cheng Kung University Medical College and Hospital, Tainan 70101, Taiwan; 2 Institute of Basic Medical Sciences, National Cheng Kung University Medical College, Tainan 70101, Taiwan; 3 Institute of Clinical Medicine, National Cheng Kung University Medical College, Tainan 70101, Taiwan; 4 Center of Infectious Disease and Signaling Research, National Cheng Kung University, Tainan 70101, Taiwan; 5 Department of Biochemistry and Molecular Biology, Medical University of South Carolina, Charleston, South Carolina 29425, USA; 6 Department of Microbiology and Immunology, National Cheng Kung University Medical College, Tainan 70101, Taiwan

## Abstract

**Introduction:**

Community-acquired pneumonia (CAP) requiring intensive care unit (ICU) treatment commonly causes acute respiratory failure with high mortality. Kallistatin, an endogenous tissue kallikrein inhibitor, has been reported to be protective in various human diseases. The aim of this study was to assess the correlations of kallistatin with other biomarkers and to determine whether kallistatin levels have a prognostic value in severe CAP.

**Methods:**

Plasma samples and clinical data were prospectively collected from 54 patients with severe CAP requiring ICU admission. Seventeen healthy control subjects were included for comparison. Plasma kallistatin, kallikrein, and other biomarkers of inflammation (tumor necrosis factor-α (TNF-α), interleukin (IL)-1β, IL-6, IL-8, C-reactive protein (CRP)), and anti-coagulation (protein C, anti-thrombin III) were measured on days 1 and 4 of ICU admission. Comparison between survivors (*n *= 41) and nonsurvivors (*n *= 13) was performed.

**Results:**

Plasma kallistatin was significantly consumed in severe CAP patients compared with healthy individuals. Lower day 1 kallistatin levels showed a strong trend toward increased mortality (*P *= 0.018) and higher day 1 CURB-65 scores (*P *= 0.004). Plasma kallistatin levels on day 1 of ICU admission were significantly decreased in patients who developed septic shock (*P *= 0.017) and who had acute respiratory distress syndrome (*P *= 0.044). In addition, kallistatin levels were positively correlated with anti-thrombin III and protein C and inversely correlated with IL-1β, IL-6, and CRP levels. In a multivariate logistic regression analysis, higher day 1 CURB-65 scores were independent predictors of mortality (odds ratio = 29.9; *P *= 0.009). Also, higher day 1 kallistatin levels were independently associated with a decreased risk of death (odds ratio, 0.1) with a nearly significant statistical difference (*P *= 0.056). Furthermore, we found that a cutoff level of 6.5 μg/ml of day 1 kallistatin determined by receiver operating characteristic curves could be used to distinguish between patients who survived in 60 days and those who did not.

**Conclusions:**

These results suggest that kallistatin may serve as a novel marker for severe CAP prognosis and may be involved in the pathogenesis of CAP through antiinflammatory and anticoagulation effects.

See related letter by Katz *et al., *http://ccforum.com/content/17/2/429

## Introduction

Community-acquired pneumonia (CAP) is a common disorder that is potentially life threatening despite the advent of potent antibiotics. Up to 36% of hospitalized CAP patients require admission to the intensive care unit (ICU). These patients present with more severe disease, more morbidities, and higher mortality rate [1]. Thus outcome evaluation is important for management of patients with severe CAP requiring ICU admission. Practice guidelines based on severity-assessment tools, such as the CURB-65 score, allow high-risk patients to be identified and given specific treatment [2]. However, the prediction rule is derived from clinical data and laboratorial parameters, which are more time-consuming and difficult to obtain, thereby limiting the clinical applications of predictive outcomes. Several biomarkers have been proposed to assess illness severity and outcome. For example, inflammatory cytokines such as interleukin (IL)-6 [3] and D-dimer [4] are associated with 30-day mortality and major morbidity in patients with CAP. Procalcitonin [5-7], albumin, and C-reactive protein (CRP) levels [8,9] have also been shown to predict the severity of illness and 28-day mortality. However, most of these factors merely reflect individual coagulation and inflammation status, but have no therapeutic potential in themselves. Therefore, identification of a new biomarker, capable not only of predicting outcomes but also of providing a potential therapeutic target, would be more useful in clinical applications.

Kallistatin, an endogenous human serine proteinase inhibitor, was originally known as a tissue kallikrein inhibitor. It binds strongly to tissue kallikrein and is able to inhibit tissue kallikrein kininogenase and amidolytic activities *in vitro *[10,11]. However, kallistatin has been reported to have various effects as an antiangiogenic, antioxidant, antiapoptotic, and antiinflammatory agent, independent of kallikrein inhibition [12-14]. A significantly reduced kallistatin level was seen in plasma samples from patients with liver disease and with sepsis, suggesting that kallistatin is produced mostly in the liver and can be consumed during sepsis. Its consumption in sepsis may indicate a protective role to prevent blood pressure reduction [10]. In an animal study, transgenic mice overexpressing rat kallikrein-binding protein, sharing a high degree of sequence identity with human kallistatin, were more resistant to LPS-induced lethality [15]. Studies investigating adenovirus-mediated kallistatin gene therapy in rat models of collagen-induced arthritis and osteoarthritis also showed that kallistatin suppresses arthritis progression through its antiangiogenesis, antiinflammation, and antiapoptosis activities [16,17]. In addition, previous studies demonstrated that kallistatin inhibits inflammatory cell infiltration and oxidative stress in animal models of myocardial ischemia-reperfusion injury, myocardial infarction, and salt-induced renal injury [13,14,18]. In the carbon tetrachloride-induced liver-injury mouse model, transgenic expression of kallistatin was shown to attenuate liver damage through reduction of oxidative stress [19]. These findings indicate that kallistatin has protective effects against vascular and organ damage by preventing inflammation, apoptosis, and oxidative stress. Given that kallistatin can be found in a wide range of human tissues and fluids, including kidney, lung, myocardium, blood vessels, plasma, and urine [10,20], its plasma levels might be relevant to infectious diseases, which commonly induce multiple organ dysfunction and lead to death. To our knowledge, clinical studies investigating the association of plasma kallistatin levels with human infectious diseases, such as CAP, are still lacking.

In this study, we hypothesized that kallistatin may be involved in regulation of the processes of inflammation and coagulation in severe CAP and may be associated with the outcome. Therefore, we sought to determine the correlations of plasma kallistatin with other biomarkers, including factors of anticoagulation (protein C, anti-thrombin III) and inflammation (tumor necrosis factor (TNF)-α, IL-1β, IL-6, IL-8, CRP), and to evaluate its prognostic value further in patients with severe CAP.

## Materials and methods

### Study population

In a prospective observational study, we consecutively enrolled patients who were admitted to our medical ICU because of severe CAP in National Cheng Kung University Hospital, a tertiary referral center in southern Taiwan, between April 2010 and January 2011. Study approval was obtained from the Institutional Review Board of the National Cheng Kung University Hospital. Written informed consent was obtained from all participants or their legal representatives if the patients were unconscious. Adult patients with pneumonia that required ICU admission were initially screened for severe CAP. Exclusion criteria were pregnancy, human immunodeficiency virus infection, immunosuppressive treatment, and tuberculosis. Pneumonia was diagnosed if patients presented with acute lower respiratory tract infection, new or progressive pulmonary infiltrates on chest radiography, and identification of microbes in the lower respiratory tract. Severe CAP was defined according to the American Thoracic Society (ATS) criteria [21]. Exclusion criteria were refusal of informed consent, prior hospitalization within 15 days before admission, pneumonia developing during hospitalization, terminal illness receiving palliative treatment, and delayed ICU admission for more than 48 hours. All patients were initially treated with broad-spectrum antibiotics, based on the ATS guideline, as early as possible [21]. Subsequent adjustments of the antimicrobial regimen were based on the antibiotic susceptibilities and the opinions of infectious specialists once causative pathogens were defined. Clinical response of antimicrobial therapies was evaluated daily, and treatments and cultures were reassessed and reexamined in patients who showed a deteriorated condition.

We also studied 17 healthy subjects for comparison analyses.

### Study design and definitions

Demographic data (age, gender, comorbidities), complications (septic shock at ICU admission, development of ARDS, need for mechanical ventilation), clinical variables (blood pressure, body temperature, respiratory rate, heart rate), laboratory data (arterial blood gas, blood cell count, biochemical data), and outcome data (length of ICU and hospital stay, duration of mechanical ventilation, hospital mortality) were collected. The worst daily values for all variables of interest were recorded to calculate the Acute Physiology and Chronic Health Evaluation II (APACHE II) score [22] on day 1 and Sequential Organ Failure Assessment (SOFA) score [23] on days 1 and 4 of ICU admission. Severe CAP was diagnosed according to ATS criteria, and its severity was evaluated by using the CURB-65 score, an acronym for each of the risk factors measured, including confusion of new onset (defined as an abbreviated mental test score of 8 or less), urea greater than 7 m*M *(19 mg/dl), respiratory rate of 30 breaths per minute or greater, blood pressure less than 90 mm Hg systolic or diastolic blood pressure 60 mm Hg or less, and age 65 years or older [21]. Patients were followed up until death or 60 days after ICU admission for outcome.

### Plasma biomarker measurements

Blood samples were collected on days 1 and 4 of ICU admission in heparinized tubes and centrifuged for 10 minutes at 3,000 *g*. Then plasma aliquots were stored at -80°C until the time of analysis. Levels of kallistatin, kallikrein, and the inflammatory biomarkers, including TNF-α, IL-1β, IL-6, and IL-8, were determined in duplicate by using enzyme-linked immunosorbent assay kits (R&D Systems, Minneapolis, MN, USA). Anticoagulation factors protein C and antithrombin III were also measured by using chromogenic assay kits (Instrumentation Laboratory, Bedford, MA, USA), and CRP by using an immunoturbidimetric method with a commercially available test (Beckman Coulter, Fullerton, CA, USA).

### Statistical analysis

All statistical analyses were performed by using a statistical software package (PASW for Windows, version 18.0; SPSS Inc, Chicago, IL, USA). Continuous variables were expressed as median (range). Differences of continuous variables between groups were compared by using the Mann-Whitney *U *test, and those of categoric variables were compared with the χ^2 ^test. The Kruskal-Wallis test was used to compare variables among more than three groups. Levels of significance were expressed as *P *values. Multivariate logistic regression analysis was performed to analyze the independent risk factors for hospital mortality. Odds ratios (ORs) and 95% confidence intervals (CIs) were calculated according to the higher value of each variable, except for gender. The median values were used to discriminate between the high and low groups. For the variable gender, male gender was used. Initially, the biomarker variables (kallistatin, kallikrein, protein C, antithrombin III, TNF-α, IL-1β, IL-6, IL-8, CRP) and disease-severity scores (CURB-65 score, SOFA score, APACHE II score) on day 1 of ICU admission were analyzed by using univariate analysis. The variables that showed significant or nearly significant differences (that is, *P *< 0.2) on univariate analysis were then entered in a multivariate logistic regression model to derive the independent prognostic factors. Spearman correlation-of-rank coefficient was used to analyze correlations between kallistatin and other biomarkers. Furthermore, the areas under the receiver operating characteristic (ROC) curve and Kaplan-Meier curve were drawn for kallistatin to evaluate the ability to discriminate between patients who survived and those who died. The optimal kallistatin cutoff was defined as the value associated with the highest sum of sensitivity and specificity (Youden index). Differences between the Kaplan-Meier curves were assessed by using the log-rank test. A two-sided *P *value ≤ 0.05 was considered to be statistically significant.

## Results

Demographic and clinical characteristics of severe CAP patients

In total, 135 patients admitted to ICUs with a diagnosis of pneumonia were screened during the enrollment period, and 13 patients were screened with a refusal of informed consent, 27 with prior hospitalization within 15 days before admission, 28 with an underlying terminal illness receiving palliative treatment, and 13 with delayed ICU admission more than 48 hours were excluded, leaving 54 patients with severe CAP included in this study. The demographic data and clinical variables are summarized in Table 1. Forty-six (85%) patients required invasive mechanical ventilation on ICU admission. The overall hospital mortality in the cohort was 24.1%. Survivors and nonsurvivors were similar in terms of age, gender, comorbidities, the need for invasive mechanical ventilation, positive blood cultures, the length of ICU and hospital stay, and the duration of mechanical ventilation. More patients had acute respiratory distress syndrome (ARDS) (*P *= 0.025) and with septic shock at ICU admission (*P *= 0.051) in the group that did not survive. Markers of disease severity, including APACHE II, SOFA, and CURB-65 scores, were significantly higher in nonsurvivors compared with survivors.

**Table 1:** Comparisons between survivors and nonsurvivors of severe community-acquired pneumonia

Variables	Survivors (*n *= 41)	Nonsurvivors (*n *= 13)	*P *value
Age, year	76 (21-93)	77 (33-96)	0.142
Gender, male, *n *(%)	27 (66)	7 (54)	0.652
Comorbidities, *n *(%)			
Diabetes	8 (20)	2 (15)	1.000
Chronic kidney disease	4 (10)	1 (8)	1.000
Chronic respiratory disease	7 (17)	2 (15)	1.000
Liver cirrhosis	2 (5)	1 (8)	1.000
Malignancy	5 (12)	1 (8)	1.000
ARDS, *n *(%)	7 (17)	7 (54)	0.025^a^
Invasive mechanical ventilation, *n *(%)	33 (80)	13 (100)	0.176
Septic shock at ICU admission, *n *(%)	20 (49)	11 (85)	0.051^b^
Positive blood cultures, *n *(%)	7 (17)	4 (31)	0.285
APACHE II score, points	18 (6-36)	25 (14-40)	0.027^a^
SOFA score, points			
Day 1	6 (0-15)	14 (3-18)	0.018^a^
Day 4	5 (0-18)	8.5 (2-17)	0.045^a^
CURB-65 score, points			
Day 1	3 (2-5)	4 (3-5)	0.002^a^
Day 4	3 (0-5)	4 (1-5)	0.021^a^
ICU stay, days	9 (3-64)	13 (2-47)	0.277
Hospital stay, days	20 (9-64)	18 (2-52)	0.302
Mechanical ventilation, days	8 (0-67)	13 (2-48)	0.247

Data are presented as median (range), unless stated otherwise. APACHE II, Acute Physiology and Chronic Health Evaluation II; ARDS, acute respiratory distress syndrome; CURB-65, confusion of new onset (defined as an abbreviated mental test score of 8 or less), urea >7 m*M *(19 mg/dl), respiratory rate of 30 breaths per minute or greater, blood pressure <90 mm Hg systolic or diastolic blood pressure 60 mm Hg or less, and age 65 years or older; ICU, intensive care unit; SOFA, Sequential Organ Failure Assessment. ^a^Statistically significant. ^b^Nearly statistically significant.

### Comparison of kallistatin and other biomarkers between the groups

**Figure 1:**
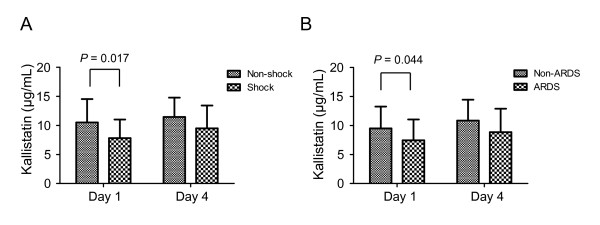
**Relation between plasma kallistatin levels on days 1 and 4 of intensive care unit (ICU) admission and the development of septic shock and acute respiratory distress syndrome (ARDS) in severe community-acquired pneumonia (CAP) patients**. Comparison of days 1 and 4 plasma kallistatin levels between patients with and without the development of septic shock **(A) **and of ARDS **(B)**. Mann-Whitney nonparametric test was used for comparisons between the groups.

We found that kallistatin was significantly consumed in CAP patients compared with healthy subjects, but no significant differences in kallikrein levels were observed between the two groups (Table 2). The comparison of plasma biomarker levels between survivors and nonsurvivors is shown in Table 3. Lower day 1 and 4 levels of kallistatin (*P *= 0.049 and *P *= 0.002, respectively) and antithrombin III (*P *= 0.037 and *P *= 0.004, respectively) and day 4 of protein C (*P *= 0.013) were found in patients who died compared with those who survived, indicating greater consumption of these factors in the group of patients who died. Nevertheless, inflammatory biomarkers, IL-8 levels on days 1 and 4 (*P *= 0.005 and *P *= 0.003, respectively), and IL-6 on day 4 (*P *= 0.052), were higher in nonsurvivors compared with survivors. No significant differences were noted in the levels of kallikrein, TNF-α, IL-1β, and CRP between survivors and nonsurvivors. In addition, we further examined the associations of kallistatin with the development of complications, and found that day 1 kallistatin plasma levels were significantly lower in patients who had septic shock and developed ARDS than those who did not (*P *= 0.017 and *P *= 0.044, respectively) (Figure 1).

**Table 2:** Comparison of plasma kallistatin and kallikrein levels between patients with severe community-acquired pneumonia and healthy individuals

	Normal subjects (*n *= 17)	Patients (*n *= 54)	
		
		Day 1	Day 4
Kallistatin (μg/ml)	17.2 (5.3-82.7)	8.3 (1.3-17.3)^a^	11.0 (1.8-18.3)^b^
Kallikrein (pg/ml)	276.9 (0-1,494.5)	203.4 (16.5-4,459.9)	164.3 (8.6-3,307.4)

Data are presented as median (range). ^a^Day 1 plasma kallistatin levels of patients were compared with those of normal subjects; *P *< 0.001. ^b^Day 4 plasma kallistatin levels of patients were compared with those of normal subjects; *P *< 0.001.

**Table 3:** Comparisons of plasma biomarkers between survivors and nonsurvivors of severe community-acquired pneumonia

Variables	Survivors (*n *= 41)	Nonsurvivors (*n *= 13)	*P *value
Kallistatin (μg/ml)			
Day 1	8.7 (3.0-17.3)	6.5 (1.3-16.2)	0.049^a^
Day 4	11.7 (2.1-18.3)	7.3 (1.8-11.3)	0.002^a^
Kallikrein (pg/ml)			
Day 1	216.6 (29.9-4,459.9)	172.0 (16.5-4,085.0)	0.436
Day 4	169.9 (8.6-3,103.1)	126.8 (50.4-3,307.4)	0.738
Protein C (%)			
Day 1	57.6 (11.3-104.0)	58.0 (19.4-251.4)	0.485
Day 4	76.0 (18.9-126.6)	54.7 (15.5-78.0)	0.013^a^
Antithrombin III (%)			
Day 1	76.0 (30.8-122.6)	62.5 (22.1-86.2)	0.037^a^
Day 4	84.0 (34.4-112.6)	60.8 (27.0-87.2)	0.004^a^
TNF-α (pg/ml)			
Day 1	4.2 (0.7-497.1)	7.7 (1.1-249.2)	0.307
Day 4	9.1 (1.2-395.9)	20.4 (2.0-2,630.8)	0.100
IL-1β (pg/ml)			
Day 1	0.6 (0.0-5.5)	0.8 (0.1-12.1)	0.390
Day 4	0.7 (0.0-14.1)	1.1 (0.5-2.3)	0.127
IL-6 (pg/ml)			
Day 1	78.1 (5.5-600.0)	197.7 (7.4-600.0)	0.108
Day 4	53.0 (3.2-257.6)	181.2 (17.3-600.0)	0.052
IL-8 (pg/ml)			
Day 1	25.2(2.4-592.0)	69.7 (18.3-4,000.0)	0.005^b^
Day 4	18.0 (2.5-160.5)	60.4 (20.8-907.0)	0.003^b^
CRP (μg/ml)			
Day 1	124.9 (9.7-433.0)	113.9 (42.9-339.5)	0.833
Day 4	57.6 (7.0-229.5)	127.6 (21.7-313.4)	0.148

Data are expressed as median (range). CRP, C-reactive protein; IL, interleukin; TNF-α, tumor necrosis factor-α. ^a^Significantly lower in nonsurvivors. ^b^Significantly higher in nonsurvivors.

### Univariate and multivariate analysis of baseline severity scores and biomarkers

**Figure 2:**
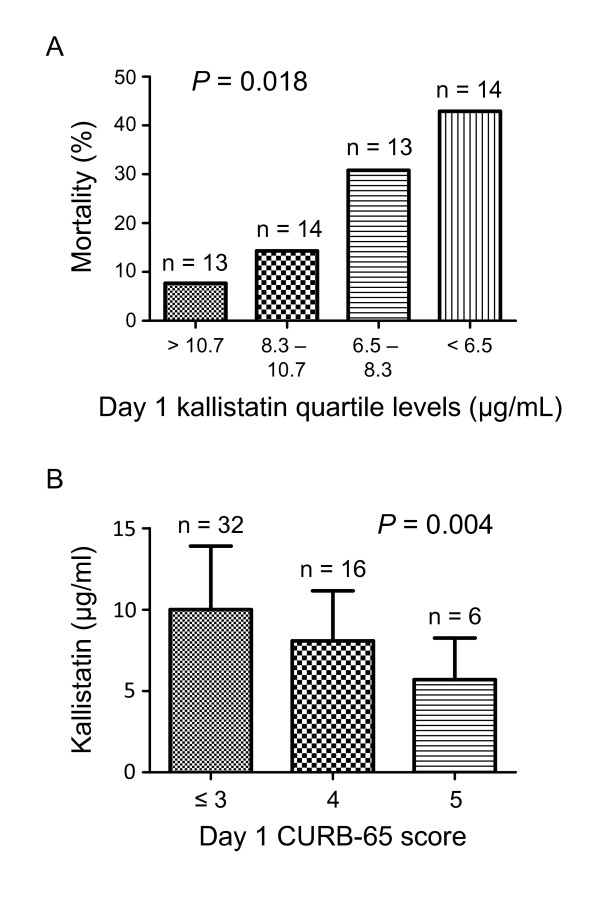
**Plasma kallistatin levels on day 1 of intensive care unit (ICU) admission are associated with the mortality and severity of community-acquired pneumonia (CAP)**. **(A) **Observed mortality according to quartile of day 1 plasma kallistatin levels. Test for trend across quartile was performed. **(B) **Plasma kallistatin levels on day 1 of ICU admission according to day 1 CURB-65 score in tertiles. Test for trend across tertiles was performed.

**Figure 3:**
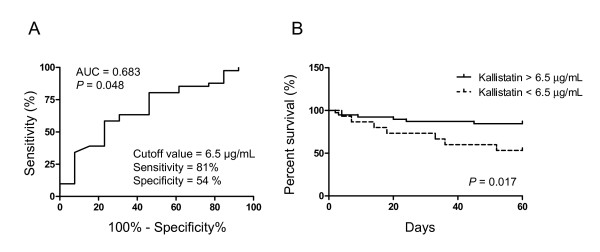
**Plasma kallistatin levels on day 1 of intensive care unit (ICU) admission and likelihood of 60-day survival**. **(A) **Receiver operating characteristic curves determining the cutoff value of day 1 kallistatin (6.5 μg/ml) to discriminate between survivors and nonsurvivors. **(B) **Kaplan-Meier curves of 60-day survival with patients grouped according to day 1 kallistatin levels >6.5 μg/ml or <6.5 μg/ml at ICU admission. Log-rank test was performed for comparisons between the groups. AUC, area under the curve.

We divided each variable into the high and low groups by using the median values, except for the variable gender. ORs and 95% CIs were calculated according to the higher value of each variable, except for gender, for which male was used. In univariate analysis, day 1 plasma kallistatin (OR = 0.21; 95% CI = 0.05 to 0.89; *P *= 0.034), IL-8 (OR = 4.71; 95% CI = 1.12 to 19.70; *P *= 0.034), CURB-65 (OR = 8.06; 95% CI = 1.88 to 34.52; *P *= 0.005), and SOFA scores (OR = 4.71; 95% CI = 1.12 to 19.70; *P *= 0.034) were significantly associated with death (Table 4). In multivariate models, apart from CURB-65 score as an independent predictor (OR = 29.85; 95% CI = 2.36 to 378.25; *P *= 0.009), the kallistatin levels tended to be associated with death among the other tested factors, with *P *< 0.2 in univariate analysis (OR = 0.11; 95% CI = 0.01 to 1.06; *P *= 0.056) (Table 4). Furthermore, we summarized the observed mortality by quartile of day 1 kallistatin levels. In comparison with the overall mortality of 24.1%, a categoric increase in death was observed from 8% in the lowest quartile to 43% in the highest quartile, with a significant test for trend across quartiles (*P *= 0.018) (Figure 2A). On further analysis of the association between day 1 kallistatin levels and severity of illness, we found a categoric decrease in kallistatin levels with a significant trend test across three categories of CURB-65 score (*P *= 0.004) (Figure 2B). In addition, to determine the optimal cutoff values of day 1 plasma kallistatin for mortality, ROC curves were also constructed with an area under the curve of 0.683 (*P *= 0.048) (Figure 3A). Optimum cutoff value of 6.5 μg/ml was obtained with the sensitivity of 81% and specificity of 54% (Figure 3A). In accordance with the cut-off value determined by the ROC curves for mortality, the 60-day survival rate was further evaluated by Kaplan-Meier analysis. All patients who did not survive in 60-day follow-up were shown to die during hospitalization, and their death was related to severe CAP, based on the medical records. Survival rate in patients with high levels of plasma kallistatin was significantly higher than in groups with low levels (Figure 3B; see also Additional file 1, Figure S1 for a large illustration).

**Table 4:** Univariate and multivariate analysis for predicting mortality in patients with severe community-acquired pneumonia

Variables	*P *value	OR	95% CI
Univariate analysis			
Age >76 years	0.436	1.647	0.470-5.778
Gender (male)	0.437	0.605	0.170-2.148
Kallistatin >8.3 μg/ml	0.034^a^	0.213	0.051-0.890
Kallikrein >203.4 pg/ml	0.343	1.853	0.518-6.629
Protein C >57.6%	0.991	1.008	0.288-3.521
Antithrombin III >72.9%	0.170	0.386	0.099-1.501
TNF-α >4.2 pg/ml	0.869	1.111	0.318-3.881
IL-1β >0.6 pg/ml	0.343	1.853	0.518-6.629
IL-6 >90.2 pg/ml	0.343	1.853	0.518-6.629
IL-8 >33.4 pg/ml	0.034^a^	4.706	1.124-19.704
CRP >129.5 μg/ml	0.750	0.816	0.234-2.851
CURB-65 score >3	0.005^a^	8.056	1.880-34.516
SOFA score >6	0.034^a^	4.706	1.124-19.704
APACHE II score >19	0.161	2.500	0.694-9.005
Multivariate analysis			
Kallistatin >8.3 μg/ml	0.056^b^	0.114	0.012-1.058
Antithrombin III >72.9%	0.457	2.262	0.264-19.390
IL-8 (pg/ml) >33.4 pg/ml	0.130	6.854	0.568-82.733
CURB-65 score >3	0.009^b^	29.850	2.356-378.246
SOFA score >6	0.589	1.760	0.226-13.684
APACHE II score >19	0.182	0.213	0.0220-2.067

APACHE II, Acute Physiology and Chronic Health Evaluation II; CI, confidence interval; CRP, C-reactive protein; CURB-65, confusion of new onset (defined as an abbreviated mental test score of 8 or less), urea >7 m*M *(19 mg/dl), respiratory rate of 30 breaths per minute or greater, blood pressure <90 mm Hg systolic or diastolic blood pressure 60 mm Hg or less, and age 65 years or older; IL, interleukin; OR, odds ratio; TNF-α, tumor necrosis factor-α. ^a^Statistically significant in univariate analysis. ^b^Statistically nearly significant or significant in multivariate analysis.

### Relation between plasma kallistatin and other biomarkers in severe CAP

**Figure 4:**
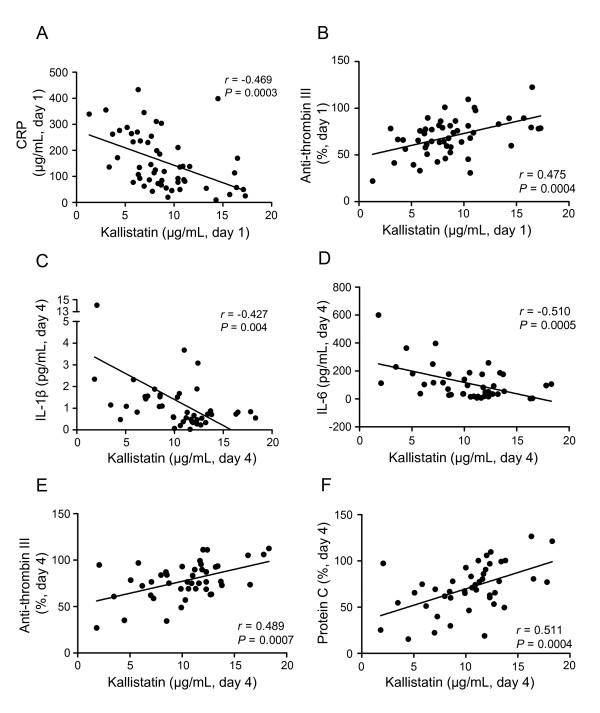
**Correlation between plasma kallistatin levels and other biomarkers in patients with severe community-acquired pneumonia (CAP)**. Correlations between plasma kallistatin levels on day 1 of intensive care unit (ICU) admission and day 1 plasma levels of C-reactive protein (CRP) **(A) **and antithrombin III **(B) **are shown. The correlations between plasma kallistatin levels on day 4 of ICU admission and day 4 plasma levels of IL-1β **(C)**, IL-6 **(D)**, anti-thrombin III **(E)**, and protein C **(F) **also are shown. Nonparametric Spearman rank-correlation coefficient was used to test correlations between two biomarkers.

Given the fact that both inflammation and coagulation have a predictive role in the severity and outcome of CAP [3,9,24,25], we further determined the association between the kallistatin and inflammation and anticoagulation factors. We found that day 1 kallistatin was negatively correlated with day 1 CRP (*r *= -0.469; *P *= 0.0003) (Figure 4A), but positively correlated with day 1 antithrombin III (*r *= 0.475; *P *= 0.0004) (Figure 4B). Furthermore, day 4 kallistatin was shown to be negatively correlated with day 4 IL-1β (*r *= -0.427; *P *= 0.004) and IL-6 (*r *= -0.510; *P *= 0.0005) (Figure 4C, D), but positively correlated with day 4 antithrombin III (*r *= 0.489; *P *= 0.0007) and protein C (*r *= 0.511; *P *= 0.0004) (Figure 4E, F).

## Discussion

This study demonstrates that plasma kallistatin is consumed in patients with severe CAP requiring ICU admission as compared with healthy individuals, and its levels are associated with disease severity and outcome. The hospital mortality rate of 24% in this study is consistent with that reported in other studies [9]. We showed that lower levels of kallistatin have a strong trend toward predicting worse clinical outcomes, accompanied by increased systemic coagulation and inflammation. We also found that kallistatin correlated positively with anticoagulation factors (protein C and antithrombin III) and negatively with inflammatory factors (CRP, IL-1β, and IL-6), suggesting that both anticoagulation and antiinflammation events may contribute to these physiological and outcome benefits. To our knowledge, this is the first study to demonstrate the role of kallistatin in severe CAP.

Kallistatin is known to play an important role in prevention of various diseases, including cancer, cardiovascular diseases, and arthritis, through the effects of antiangiogenic, antiinflammatory, antiapoptotic and antioxidative processes [12,13,16,18]. A prior study of kallistatin in human sepsis showed significantly reduced plasma kallistatin levels in patients with sepsis compared with healthy individuals, suggesting a protective role of kallistatin in sepsis through regulation of inflammatory processes [10]. In a murine model of endotoxic shock, transgenic mice overexpressing the rat kallikrein-binding protein gene, a functional analogue of kallistatin, were shown to have a higher survival rate than nontransgenic control mice [15]. Our study demonstrated similar results with decreased plasma kallistatin levels at days 1 and 4 of ICU admission in severe CAP patients compared with healthy individuals. Kallikrein is a component of the kallikrein/kinin system, which is known to regulate hemostatic and inflammatory processes and can be consumed during sepsis [26]. However, we found that plasma kallikrein levels were not significantly different between CAP patients and healthy individuals. The discrepancy may be related to the differences in studied populations or reflect the relatively small sample size. Of note, no correlations were found between kallistatin and kallikrein in our study, suggesting that the effects of kallistatin were independent of the kallikrein/kinin system. In addition, lower plasma kallistatin levels were found in patients affected with septic shock and ARDS, suggesting the protective effects of kallistatin against such complications.

In agreement with other reports in different populations, severity-score systems showed an association with mortality in CAP [3,7,27]. As expected, the SOFA and CURB-65 were associated with hospital mortality. In keeping with prior studies [3,24,28,29], we demonstrated that IL-8 levels were higher in nonsurvivors, whereas protein C and antithrombin III levels were lower in patients who died. These findings indicate that greater upregulation of the acute inflammatory process and activation of coagulation pathways occurred in patients with severe CAP who did not survive their illness. We also showed lower kallistatin levels in the nonsurvivors than in the survivors. However, kallikrein levels did not differ between the two groups, implying that the potential effects of kallistatin on the outcomes of CAP are not related to the interaction with kallikrein.

In multivariate analysis for biomarkers and severity scores, CURB-65 was shown to be an independent predictor for mortality in keeping with other reports [3,7,27]. Although we found only a nearly statistical significance in kallistatin to predict mortality independently, we indeed demonstrated that decreased plasma kallistatin levels have a strong trend toward increased mortality and severity. These findings were also confirmed by a Kaplan-Meier survival-curve analysis, which revealed a better survival rate in patients with high kallistatin levels than in those with low levels.

We observed that lower kallistatin was associated with a reduced anticoagulation, shown by decreased levels of antithrombin III and protein C, but associated with increased inflammation, demonstrated by elevated levels of IL-1β, IL-1, and CRP. These findings are supported by other studies that showed markedly lower circulating kallistatin levels in humans with sepsis [10] and with necrotic acute pancreatitis [30], in which the inflammatory response is enhanced, and the anticoagulant response is suppressed [29]. In animal studies, kallistatin gene delivery inhibited inflammation in a rat renal disease model [14], acute myocardial ischemia-reperfusion injury [13], and a rat arthritis model [16], indicating that kallistatin is relevant to an antiinflammatory effect. To the best of our knowledge, no reports illustrate the relation between kallistatin and anticoagulant factors. Although it is well known that the kallikrein/kinin system is involved in regulation of hemostasis via activation of the intrinsic pathway of coagulation, leading to the formation of a fibrin network, the role of this system in induction of pathologic coagulation disorders in severe infectious diseases is still controversial [26]. Another possible reason is that declining circulating antithrombin III and protein C levels caused by increased consumption during illness may simply be a marker for greater severity of illness, as were lower kallistatin levels.

A strength of this study was the concurrent measurement of other biomarkers, including inflammation and anticoagulant factors involved in the pathogenesis of CAP, and evaluated the relations between kallistatin and these molecules. The major limitations of our study include its small sample size in a single medical center, which may account for some lack of statistical significance. The small patient population also prevented a subgroup analysis to examine further the differences in the effects of kallistatin on clinical outcomes according to some comorbidities. However, the distribution pattern of comorbidities between survivors and nonsurvivors was similar in our cohort. Furthermore, we cannot exclude the possibility that different therapeutic interventions may affect the results through unknown interactions with kallistatin. Future studies using more subjects are required to substantiate our findings further and to confirm the potential benefits of kallistatin in severe CAP.

## Conclusions

In this study, we demonstrated that lower levels of kallistatin are associated with more-severe illness and increased mortality. In addition, kallistatin levels were positively correlated with the levels of anticoagulation factors (antithrombin III and protein C) and inversely correlated with the levels of inflammatory mediators (IL-1β, IL-6, and CRP). These findings indicate that kallistatin may be protective against severe CAP, which implies possible therapeutic benefits of kallistatin in these patients. Follow-up animal studies would be useful to confirm our observations.

## Key messages

• Plasma kallistatin was consumed more in patients with severe community-acquired pneumonia (CAP) than in healthy individuals.

• Lower plasma kallistatin levels on day 1 of ICU admission were correlated with increased in-hospital and 60-day mortality and elevated day 1 CURB-65 scores in patients with severe CAP.

• Lower day 1 plasma kallistatin levels were associated with the development of septic shock and acute respiratory distress syndrome in patients with severe CAP.

• Plasma kallistatin levels were positively correlated with the levels of antithrombin III and protein C and negatively correlated with the levels of IL-1β, IL-6, and CRP.

• The results indicate that plasma kallistatin was associated with the outcomes of severe CAP, which may be related to antiinflammatory and anticoagulative effects.

## Abbreviations

APACHE: Acute Physiology and Chronic Health Evaluation; ATS: American Thoracic Society; ARDS: acute respiratory distress syndrome; AUC: area under the curve; CAP: community-acquired pneumonia; CRP: C-reactive protein; CI: confidence interval; IL: interleukin; CURB-65: confusion of new onset (defined as an abbreviated mental test score of 8 or less), urea greater than 7 m*M *(19 mg/dl), respiratory rate of 30 breaths per minute or greater, blood pressure less than 90 mm Hg systolic or diastolic blood pressure of 60 mm Hg or less, and age 65 years or older; ICU: intensive care unit; OR: odds ratio; ROC: receiver operating characteristic; SOFA: Sequential Organ Failure Assessment; TNF-α: tumor necrosis factor-α.

## Competing interests

All the authors declare that they have no competing interests.

## Authors' contributions

WCL participated in the study design, performed biomarkers analysis, collected data, analyzed data, and wrote the manuscript. SLL carried out kallistatin analysis and analyzed data. CFL performed parts of the biomarkers analysis and analyzed data. CWC was responsible for including patients and data collection and analysis. LC participated in the study design and data analysis. JC was responsible for study design, data analysis, and manuscript revision. YSL was responsible for study design and manuscript revision. All authors read and approved the final manuscript.

## Supplementary Material

Additional file 1 **Figure S1**. **Kaplan-Meier curves of 60-day survival (large illustration)**. Click here for file
