# Supplementary figures and images for: Plasma kallistatin levels in patients with severe community-acquired pneumonia
Source: Crit Care. 2013 Feb 8;17(1):R27. doi: 10.1186/cc12507 (PMC4056893; doi:10.1186/cc12507)

Figure S1

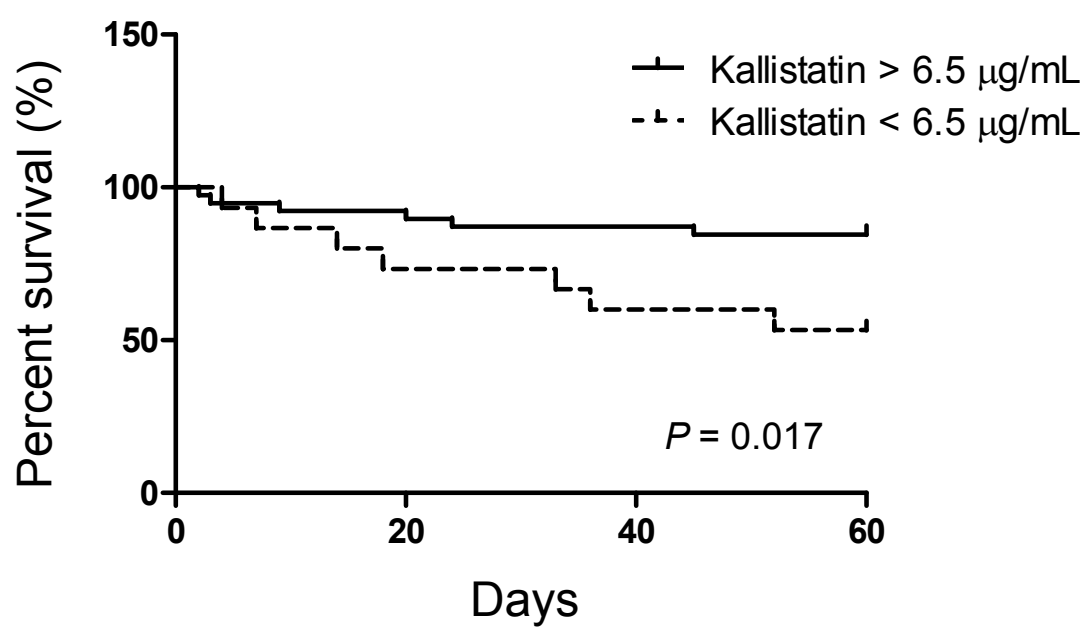

Supplement: Additional file 1 — Figure S1. Kaplan-Meier curves of 60-day survival (large illustration). [file cc12507-S1.PDF]
